# Hangings attended by emergency medical services: a scoping review

**DOI:** 10.29045/14784726.2021.3.5.4.40

**Published:** 2021-03-01

**Authors:** Gary Shaw, Lee Thompson, Graham McClelland

**Affiliations:** North East Ambulance Service NHS Foundation Trust ORCID iD: https://orcid.org/0000-0001-5279-1412; North East Ambulance Service NHS Foundation Trust; North East Ambulance Service NHS Foundation Trust

**Keywords:** cardiac arrest, emergency medical services, hanging

## Abstract

**Background:**

In the United Kingdom (UK) there were 6507 deaths by suicide in 2018, with hanging being the most common method. Hanging will normally result in emergency medical services (EMS) being called and may result in resuscitation being attempted. Trauma audits conducted by North East Ambulance Service NHS Foundation Trust have identified an increased trend in hanging cases, which were also reported in national data. The aim of this scoping review was to explore the literature around EMS attendance at hangings to inform further research and clinical practice.

**Methods:**

A five-stage scoping review method was used. Relevant studies were identified by searching MEDLINE, CINAHL, EMBASE and EMCARE with the help of the Library and Knowledge Service for NHS Ambulance Services in England. Grey literature and reference lists were also searched. Studies were included based on relevance to hangings attended by EMS. Data were tabulated and narratively synthesised.

**Results:**

Sixteen papers were included in the review. Australia was the most frequent source of studies (n = 5, 31%). Most studies (n = 11, 69%) were published in the past 10 years. The median sample size was 53 (IQR 41–988, range 10–3981). All papers included varying levels of patient characteristics, EMS input and patient outcomes.

**Conclusion:**

Hanging is a highly lethal method of suicide that is increasingly used in the UK. This scoping review found that there is scarce literature focused on hangings attended by EMS. Treatment of the hanging patient in cardiac arrest is described in many of the papers included. Hanging patients may benefit from the presence of specialist resources who can deliver interventions such as sedation and advanced airway management. The psychological impact of attending, or witnessing, hanging patients is an area that needs further consideration. Further research is needed to describe and improve EMS treatment of hangings.

## Introduction

Suicide accounted for 1.5% of deaths worldwide between 1990 and 2016, with low- and middle-income countries accounting for most cases, and is the leading cause of death among young people aged 15–24 years (Fazel & Runeson, 2020).

In the United Kingdom (UK), hanging was the most common method of completed suicide in 2018, accounting for 59% of male and 45% of female suicides, and an increasing trend in hanging, as opposed to other methods of suicide, was also reported (Office for National Statistics (ONS), 2019). As in the general population, hanging was the most common suicide method reported in ambulance service staff in recent UK (Mars et al., 2020) and Australian studies (Milner et al., 2017).

Hanging causes death by asphyxiation resulting from constriction of the neck causing obstruction of the large blood vessels in the neck leading to cerebral hypoxia (Rutty, 2016). Hanging rarely causes cervical spinal fractures (Rao, 2016). Hangings largely happen in the community, most often at home (Gunnell et al., 2005; Yau & Paschall, 2018), and therefore emergency medical services (EMS) will often be called.

Trauma audits conducted by North East Ambulance Service NHS Foundation Trust have identified an increased trend in hanging cases. Further investigation revealed that the region had the highest age standardised rate of suicide for males in 2018, with 20.4 deaths per 100,000 population against an average rate of 16.2 across England and Wales (ONS, 2019).

The aim of this scoping review was to explore the literature around EMS attendance at hangings and to identify factors needing consideration in order to develop future studies in this area and influence clinical practice.

## Methods

A scoping review was chosen in order to explore the broad topic area and map out key concepts, studies and gaps in the literature (Colquhoun et al., 2014). A five-stage scoping review method (Arksey & O’Malley, 2005) was used, supported by the PRISMA-ScR checklist (Supplementary 1) (Tricco et al., 2018).

### Stage 1: identifying the research question

The rationale and background for the research question are described in the introduction.

### Stage 2: identifying relevant studies

Relevant studies were identified by searching electronic databases, grey literature and reference lists.

- Step 2.1. Initial search using the Library and Knowledge Service for NHS Ambulance Services in England (LKS). The terms and search strategy used are in Supplementary 1.
- Step 2.2. The grey literature was searched using the first 200 results on Google Scholar (Haddaway et al., 2015).
- Step 2.3. The reference list of papers identified in the LKS search and grey literature was examined for further papers.

Studies were selected based on the following criteria.

#### Inclusion criteria

- Participants: Patients attended by EMS due to accidental or non-accidental hanging. All ages were included.
- Concept: The characteristics, treatment and outcomes of patients were the three core concepts explored in this review.
- Context: The context was defined by the involvement of EMS.

Hanging has historically been described as judicial or non-judicial. Judicial hanging is less relevant to modern UK paramedic practice as the last judicial hanging in the UK was 13 August 1964 (Capital Punishment UK, 2020), so we elected to focus on non-judicial hanging (Gubbins, 2016; Rutty, 2016).

#### Exclusion criteria

- Participants: No exclusions.
- Time period: Studies published before 2000 were excluded due to the rapid development of pre-hospital care in the past two decades.
- Type of studies: Case reports, animal studies and letters were excluded. Studies published in languages other than English were excluded.

### Stage 3: study selection

Studies were selected based on title and abstract from the initial LKS search by GM. The grey literature was searched by LT, with relevant studies identified by title and abstract. Potentially relevant studies were sourced as full-text papers and discussed by the whole study team, and a consensus was reached as to inclusion. If the three authors were unable to agree on inclusion, a majority decision was taken. A single author (GM) examined the reference list of studies selected for inclusion, and further potentially relevant papers were discussed and a collective decision was taken on inclusion.

### Stage 4: charting the data

Data were extracted from each included study using a standard form including the information listed below:

- Author(s)
- Year of publication
- Origin/country of origin
- Aims/purpose
- Study population and sample size
- Methodology
- Description of patients included
- EMS details included
- Patient outcomes included
- Key findings.

Extraction data were discussed within the team and narratively synthesised. Diagrams were used to summarise the range of concepts reported. No analysis of study quality was completed due to the use of the scoping review method.

### Stage 5: collating, summarising and reporting the results

Results are summarised and discussed below.

## Results

The initial search was completed on 22 January 2020 with the help of the LKS. MEDLINE, CINAHL, EMBASE and EMCARE were searched and returned 110 records. The Google Scholar search for grey literature was completed on 28 March 2020 and returned 34 records. Sixteen potential papers were identified from reference list searches.

**Figure 1:**
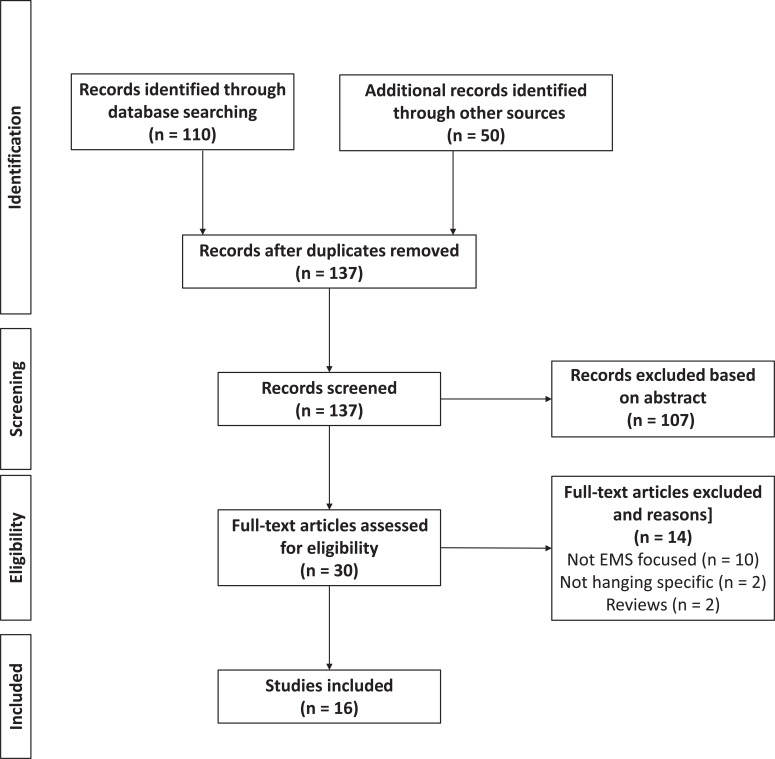
Figure 1. PRISMA-style flowchart of study selection process.

Once duplicates had been removed, 30 full-text papers were reviewed. Thirteen papers (Alqahtani et al., 2019; Atreya & Kanchan, 2015; Davies et al., 2011; Deasy et al., 2011, 2013; Escutinaire et al., 2019; Kao & Hsu, 2018; Kim et al., 2016; Martin et al., 2005; Matsuyama et al., 2004, 2016; Rehn et al., 2018; Wee et al., 2012) were included in the review based on the initial papers. A further three (Boots et al., 2006; Hanna, 2004; Penney et al., 2002) were included based on papers identified in the reference list searches. The selection process is shown in Figure 1.

The identification and screening process resulted in 16 papers being included in the review, which are summarised in Table 1. Australia was the most frequent source of studies (n = 5, 31%), and two (13%) studies were UK based. Most studies (n = 11, 69%) were published in the past 10 years. The median sample size was 53 (IQR 41–988, range 10–3981). All papers included varying levels of patient characteristics, EMS input and patient outcomes.

**Table 1.** Summary of included studies.

Lead author	Year	Country of origin	Aim/purpose	Study population and sample size	Methods	Key results
Alqahtani	2019	Australia	Assess temporal trends in incidence, characteristics and survival of hanging-related out-of-hospital cardiac arrest (OHCA)	3981 hanging-related OHCA. Mean age 38 years. 75% male (in EMS-treated patients).	Retrospective review	Incidence of hanging doubled over 18 years, driven by adults aged 18–64. EMS resuscitation rates increased from 20 to 29%. Survival rate remained stable at 3%.
Atreya	2015	Nepal	Describe near hanging cases in detail	Ten near hanging cases. Mean age 29 years. 50% male.	Retrospective review	Small sample, young population, equal number male and female, 100% survival in near hanging.
Boots	2006	Australia	Determine the epidemiology of near hanging	161 near hangings. Mean age 31 years. 82% male.	Retrospective review	Young male population with 42% previous psychiatric illness. Short hanging times reported with 1/3 in contact with ground. 1/3 required cardiopulmonary resuscitation (CPR). 43% required intubation. 16% mortality. Predictors of mortality identified.
Davies	2011	Canada	Describe prognostic markers in paediatrics following hanging or strangulation	41 children (age <18 years). Mean age 13 years. 68% male.	Retrospective review	Absence of a pulse was highly predictive of poor outcome whereas Glasgow Coma Scale (GCS) = 3 was less predictive. No c-spine injuries reported.
Deasy	2011	Australia	Describe characteristics and outcomes of paediatrics following OHCA caused by hanging	53 children (age <18 years). Median age 16 years. 59% male.	Registry review	Accidental hangings in younger children and deliberate in older children. Asystole common presenting rhythm. No c-spine injuries reported. Prevention is key intervention.
Deasy	2013	Australia	Describe characteristics and profile of adult hanging patients	1321 hanging-related OHCA. Median age 39 years. 88% male.	Registry review	Hanging = 4% of adult OHCA with young, male demographic. Bystander CPR linked to EMS resuscitation and outcome. Hanging common method of completed suicide. Resuscitation is not futile in this population.
Escutinaire	2019	France	Identify prognostic criteria in hanging-related OHCA to define termination of resuscitation rules	1689 hanging-related OHCA. Median age 48 years. 78% male.	Registry review	Identified scarcity of literature. Young, male population with 2.1% survival. Early Basic Life Support (BLS) associated with positive outcomes but may need to be prolonged attempt. CPR is not futile in this population. Unable to define termination of resuscitation criteria.
Lead author	Year	Country of origin	Aim/purpose	Study population and sample size	Methods	Key results
Hanna	2004	UK	Analyse the epidemiology, methods, outcomes and complications of near hangings	13 near hangings. Mean age 31 years. 92% male.	Retrospective review	Young male population. Poor documentation of height and estimated suspension time. Prisoners highlighted. High rate of psychiatric history and previous suicide attempts.
Kao	2018	China	Describe hanging and near hanging patients and identify prognostic factors	41 patients admitted to emergency department (ED) via EMS. Mean age 56 years. 56% male.	Unclear	Gender balance and older population. Malignancy and previous psychiatric history reported. Low GCS, pupil dilation and lack of pupillary reflexes predicted poor outcome.
Kim	2016	Korea	Explore outcomes of hanging patients and prognostic factors	280 hanging patients. Mean age 43 years. 51% male.	Retrospective review	OHCA or low GCS (coma) are predictive of poor outcome.
Martin	2005	America	Analyse epidemiology, injuries and outcomes of hanging and near hanging patients	655 hanging patients. Mean age 30 years. 84% male.	Registry review	Low GCS and low respiratory rate predictive of poor outcome. High incidence of spinal injuries. Use of alcohol (19%) and drugs (22%) reported.
Matsuyama	2004	Japan	Identify prognostic factors	47 hanging patients. Mean age 53 years. 47% male.	Unclear	Older female demographic. 32% history of psychiatric illness. High mortality rate. Hanging time and GCS3 associated with survival. Circumferential ligature marks highlighted as prognostic.
Matsuyama	2016	Japan	Assess characteristics and outcomes of self-inflicted injuries	9424 self-inflicted injuries including 1489 hangings. Average age not reported. 68% male.	Retrospective review	Hanging reported among other self-harm methods. Male demographic. Hanging was most lethal self-harm method with the lowest rate of EMS transport.
Penney	2002	Australia	Identify prognostic factors for hanging injuries	42 hanging patients. Average age not reported. 90% male.	Retrospective review	Oldest data. Young, male demographic. 50% psychiatric history. 70% drugs and/or alcohol ingestion.
Rehn	2018	UK	Describe pre-hospital management of paediatric hangings	31 paediatric (age <16 years) hangings. Median age 13 years. 71% male.	Registry review	Male suicidal intent in older children, accidental hanging in younger children. 80% intubation by physician-led team.
Wee	2012	Korea	Describe characteristics and outcomes of hanging-induced OHCA	52 hanging patients with OHCA. Mean age 49 years. 42% male.	Registry review	Hanging identified as a unique cause of cardiac arrest. 10% bystander CPR but 65% EMS CPR. 90% asystole as first rhythm.

## Discussion

**Figure 2:**
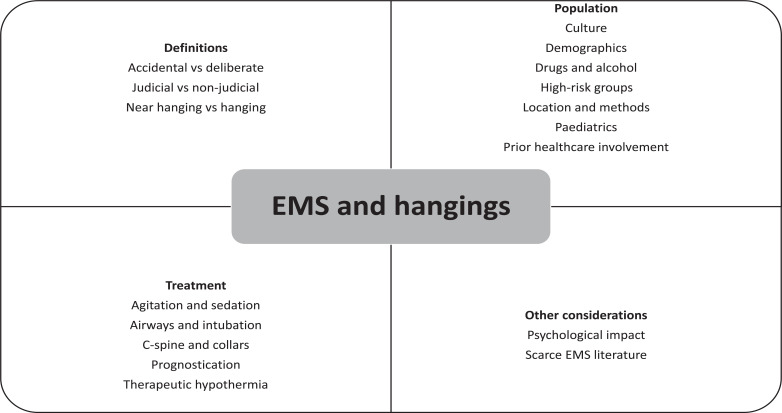
Figure 2. Topics relevant to EMS consideration of hangings.

Hanging is a highly lethal method of suicide and occasionally a tragic accident. This review identified limited literature discussing hangings attended by EMS and most of the studies included EMS and ED treatment. Three interconnected topics were identified for discussion: definition of the population; factors affecting the population; and treatment and management by EMS and beyond. A fourth topic considers psychological impact and the scarcity of EMS literature. The topics are displayed in Figure 2.

## Definitions

Older papers referred more often to judicial hangings whereas this has become less and less relevant as time has moved on. Older data on hanging pathology came from studies of judicial hangings and focused on c-spine injuries as the primary cause of death, but more recent studies have identified constriction of the blood vessels supplying the brain as the primary cause of death in non-judicial hangings (Rutty, 2016).

Near hanging is defined as ‘an unsuccessful attempt at non-judicial hanging’ (Hanna, 2004) or ‘An act of hanging where the victim survives long enough to reach the medical care facility’ (Atreya & Kanchan, 2015). This terminology is unhelpful for EMS as it does not account for resuscitation or care delivered by EMS. The authors believe that it is more relevant to EMS to define hanging in terms of whether the patient presents in cardiac arrest or not. The cardiac arrest population can also be divided into patients where resuscitation was attempted and those with no resuscitation.

Most of the literature focused on deliberate hanging as a method of suicide but there were also accidental hangings described within the literature. Accidental hangings primarily involve younger children (Davies et al., 2011; Deasy et al., 2011; Rehn et al., 2018), and known risks such as window blinds have led to public health initiatives.

## Population

Separate from but connected to the terms used to define hangings, the population, and factors affecting that population, was another key topic identified from the literature.

Three papers in the review reported incidence rate. Alqahtani et al. (2019) reported a crude incidence rate of 3.8 hanging-related cardiac arrests per 100,000 person years in Australia. Deasy et al. (2011) reported an incidence of hanging-related cardiac arrest in Australian children (age <18 years) of 4.4 per million per year. Martin et al. (2005) reported an incidence of 0.14% for hanging injuries in the American National Trauma Data Bank.

In the UK, the demographics of suicides are reported through the ONS. A recent report stated that suicide peaked in middle age (45–49 years) and then again in old age (80–84 years), and that suicide in the young (10–24 years) was increasing (ONS, 2019). The ONS also reported that hanging was the most common method of suicide, accounting for 59% of male deaths (n = 2912) and 45% of female deaths (n = 722). The papers included in this review support this male bias, with 13/16 papers reporting more male than female hangings. The demographics of hanging patients may be changing (Alqahtani et al., 2019), which is a topic needing further consideration.

However, there may be cultural differences in hanging patient demographics. When papers from Asian countries (China, Japan, Korea, Nepal) are compared with those from other countries, the Asian countries report a more balanced mix of males and females, including the only papers with more female than male hangings (Matsuyama et al., 2004). The Asian papers also described a slightly older population, with only Nepal reporting an average age below 43 years, whereas in non-Asian countries only one paper reports an average age above 39 years.

The most common place to find hanging patients was in the home (Deasy et al., 2013; Escutinaire et al., 2019; Matsuyama et al., 2016), but psychiatric hospitals and prisons were highlighted as potential locations in the papers by Boots et al. (2006) and Hanna (2004). Other environmental factors considered in the literature include the method and material used in the hanging (Boots et al., 2006). The involvement of drugs and alcohol in hangings was highlighted, which may be suspected from the scene of the incident (Martin et al., 2005; Penney et al., 2002).

Paediatrics need to be considered as a distinct population due to the very small numbers and limited literature. The three paediatric papers (Davies et al., 2011; Deasy et al., 2011; Rehn et al., 2018) reported average ages between 13 and 16, with a male bias. Accidental hangings were more frequently reported in very young children with a median age of three years, compared with a median age of 16 years in deliberate hangings reported by Deasy et al. (2011).

## Treatment

The EMS treatment and management of hangings is largely determined by whether the patient is in cardiac arrest or not. Patients who are not in cardiac arrest will be treated depending on their presentation and may need psychological assessment to explore the reason for the hanging attempt. Normal resuscitation protocols should be followed for patients in cardiac arrest but there are factors relating to hanging being the cause of the arrest such as the intentional nature of the act, the potential for substance ingestion alongside the hanging attempt and the typically younger age of the patient that need to be considered.

Deciding whether to resuscitate the patient is a decision that needs to be rapidly made. Many papers considered prognostication in hanging patients and how decisions about resuscitation could be supported and informed. Indicators of a poor outcome included: lack of bystander CPR (BCPR) (Deasy et al., 2013; Wee et al., 2012); circumferential ligature marks (Matsuyama et al., 2004); Glasgow Coma Score (GCS) of three either at the scene or at Emergency Department (ED) arrival (Boots et al., 2006; Kao & Hsu, 2018); asystole at the scene (Escutinare et al., 2019); pupil dilation and lack of responsiveness (Kao & Hsu, 2018). Contact with the ground (Boots et al., 2006) and hanging time <5 minutes were associated with positive outcomes, whereas >30 minutes hanging was associated with a negative outcome (Matsuyama et al., 2004). At the time of writing, no formal prognostic rule specific to hangings is being used in clinical practice in the UK to the best of the authors’ knowledge.

Bystander actions influence EMS actions and BCPR was a strong influence on EMS decision to resuscitate, according to Alqahtani et al. (2019). However, bystander actions may be different when finding a hanging as opposed to a cardiac arrest from a different cause, which may influence their willingness to do CPR or other interventions prior to the arrival of EMS. Deasy et al. (2013) reported 14% BCPR in hanging compared to 26% in OHCA due to presumed cardiac causes. The rate of BCPR ranged from 10% (Wee et al., 2012) to 65% (Alqahtani et al., 2019) across the included literature.

Airway management needs to be addressed and intubation may be required for hanging patients, especially as there may be damage to the neck and underlying airway structures. Intubation rates varied in the literature, with Boots et al. (2006) reporting 9% pre-hospital whereas Alqahtani et al. (2019) reported 54%. Paediatric patients were intubated more often, with 80–84% reported (Davies et al., 2011; Rehn et al., 2018). Variations in practice, and reasons for these variations, are subjects for further exploration.

The literature is conflicted as to whether collars should be applied for potential cervical spine injuries in hanging patients. The incidence of cervical spine injuries in hanging patients is reported to be low, with figures of 0–9% reported (Kim et al., 2016; Martin et al., 2005; Matsuyama et al., 2004; Penney et al., 2002). The incidence of cervical spinal injuries is potentially associated with the age of the patient and the height involved. Cervical collars have been associated with increased intracranial pressure due to venous compression which may worsen hanging patient outcomes (Sundstrøm et al., 2014). Further clarification of the benefits and risks associated with collar use in these patients is needed to inform evidence-based treatment pathways.

The increasing numbers of hangings attended by EMS may result in an increased number of survivors. While increasing the number of survivors from the initial injury is a positive step, EMS services need to be able to provide high quality care to these patients if patient outcomes are to be maximised, which may require specialist skills (e.g. sedation, intubation) that may not be widely available.

Patients who are resuscitated, or who were peri-arrest, may have cerebral hypoxia and present with agitation, confusion or seizures (Wee et al., 2012). Managing resuscitated hypoxic patients, who may have other injuries, is challenging in the pre-hospital setting and patients may benefit from sedation if available. Dispatching specialist resources, with critical care or physician responders, who can sedate and anaesthetise these patients may maximise long-term outcomes.

Therapeutic hypothermia was mentioned in a small number of more recent papers (Escutinaire et al., 2019; Kim et al., 2016; Wee et al., 2012) as a treatment used in post-arrest hanging patients. Therapeutic hypothermia and temperature management were administered in-hospital as opposed to in the pre-hospital setting, but if they prove beneficial may be an option for pre-hospital treatment.

### Other considerations

While many papers related to hanging were identified in the literature, very few were specific to EMS or related to EMS treatment of hangings. However, when reviewing the hanging literature one factor that stood out was the psychological impact that these cases can have. Hangings affect the public, in terms of bystanders and family members, and the attending EMS clinicians. Post-incident support needs to be considered for all involved. A recent study by Nelson et al. (2020) discussed the toll that attending cases such as hangings can have on EMS clinicians and the need for training and support for staff. The two papers mentioned earlier (Mars et al., 2020; Milner et al., 2017) demonstrate that there may be a potential link showing that EMS staff are at risk from hanging as a form of suicide.

### Areas for further study

Based on this review, the following EMS-related topics were identified as potential subjects for further exploration:

- Changes in frequency and demographics
- EMS treatment of the resuscitated hanging patient including whether to collar, the need to intubate, sedation provision and prognostication
- Impact of specialist care attendance at hanging cases
- Impact of hangings on attending clinicians and members of the public.

## Limitations

This study was a scoping review so there was not the same level of critical appraisal of papers or assessment of biases as in a systematic review. Many of the papers included in the review described hanging patients who were transported to EDs and included some EMS data among in-hospital data. This highlights the scarcity of EMS-focused literature but also introduces a selection bias as many of these papers do not consider hanging patients attended by EMS who were not transported.

## Conclusions

Hanging is a highly lethal method of suicide that is increasingly used in the UK. This scoping review found that there is scarce literature focused on hangings attended by EMS. Hangings are described using various definitions, but we believe describing hangings as resulting in cardiac arrest or not resulting in cardiac arrest is most useful for EMS. The population of hanging patients varies by region but in the UK is largely middle aged and male. Treatment of the hanging patient in cardiac arrest is described in many of the papers included, and hanging patients may benefit from the presence of specialist resources who can deliver interventions such as sedation and advanced airway management. The psychological impact of attending, or witnessing, hanging patients is an area that needs further consideration. Further research is needed to describe and improve EMS treatment of hangings.

## Acknowledgements

Thanks to the LKS for their support. Thanks to Wilma Harvey-Reid for her critical review.

## Author contributions

GS came up with the concept for this study. LT and GM supported the scoping review. All authors were involved in all stages of the review and the drafting and approval of the final manuscript. GS acts as the guarantor for this article.

## Conflict of interest

GM is on the editorial board of the BPJ.

## Funding

None.
